# *Raoultella planticola* bacteremia-induced fatal septic shock following burn injury

**DOI:** 10.1186/s12941-018-0270-0

**Published:** 2018-05-04

**Authors:** Tetsuya Yumoto, Hiromichi Naito, Hiromi Ihoriya, Kohei Tsukahara, Tomoyuki Ota, Toshiyuki Watanabe, Atsunori Nakao

**Affiliations:** 1 Advanced Emergency and Critical Care Medical Center, Okayama University Hospital, 2-5-1 Shikata-cho, Kita-ku, Okayama-shi, Okayama 700-8558 Japan; 2 Department of Plastic and Reconstructive Surgery, Okayama University Graduate School of Medicine, Dentistry and Pharmaceutical Sciences, 2-5-1 Shikata-cho, Kita-ku, Okayama-shi, Okayama 700-8558 Japan

**Keywords:** Burn, Sepsis, *Raoultella planticola*

## Abstract

**Background:**

*Raoultella planticola*, a Gram-negative, aerobic bacillus commonly isolated from soil and water, rarely causes invasive infections in humans. Septic shock from *R. planticola* after burn injury has not been previously reported.

**Case presentation:**

A 79-year-old male was admitted to the emergency intensive care unit after extensive flame burn injury. He accidently caught fire while burning trash and plunged into a nearby tank filled with contaminated rainwater to extinguish the fire. The patient developed septic shock on day 10. The blood culture detected *R. planticola*, which was identified using the VITEK-2 biochemical identification system. Although appropriate antibiotic treatment was continued, the patient died on day 12.

**Conclusions:**

Clinicians should be aware of fatal infections in patients with burn injury complicated by exposure to contaminated water.

## Background

*Raoultella planticola* is a Gram-negative, aerobic bacillus commonly found in soil and aquatic environments [1]. *R. planticola* infection is a relatively uncommon clinical entity, but sometimes causes fatal infection in immunocompromised patients [2]. Contaminated water used to extinguish a fire can lead to severe wound infections caused by waterborne microorganisms [3]. To our knowledge, *R. planticola* bacteremia has not been previously described in a burn patient. Herein, we report a case of fatal septic shock due to *R. planticola* bacteremia following flame burn injury complicated by exposure to contaminated water to extinguish the fire.

## Case presentation

A 79-year-old male was referred to our tertiary hospital after extensive flame burn injury. He accidently caught fire while burning trash in a steel drum. The fire was extinguished when he plunged into a nearby water storage tank filled with dirty rainwater. His medical history was unremarkable except for hemiparesis of the right side as sequelae of ischemic stroke.

**Fig. 1:**
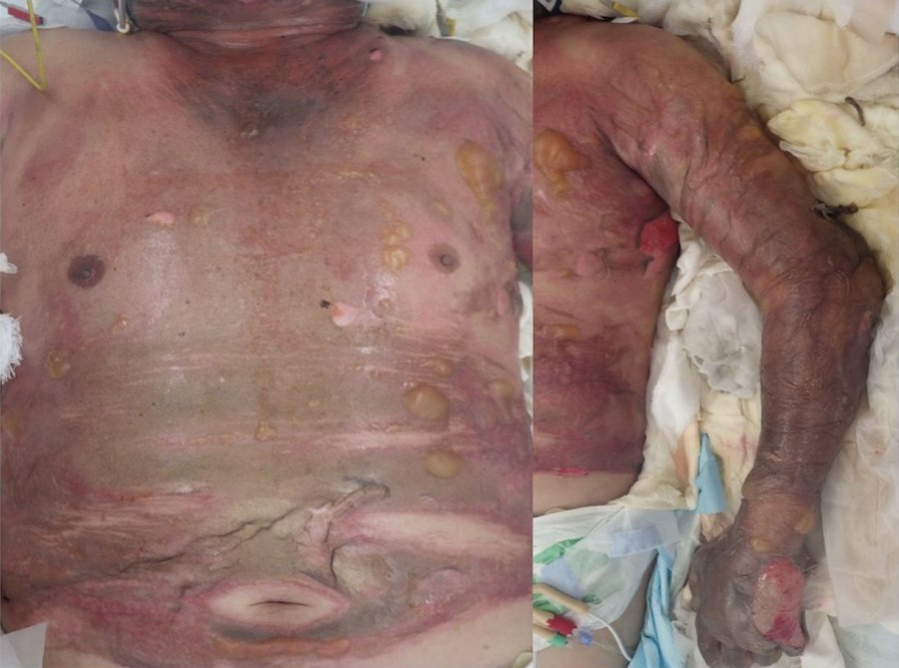
The patient at admission. Burn wounds were distributed on the face, neck, anterior trunk, and both upper extremities. The total body surface area affected was 35, 30% with third-degree burns and 5% with eventual deep dermal burns

Upon arrival in the emergency department, he was alert and oriented, but he appeared distressed, complaining of dyspnea. His vital signs were as follows: Glasgow Coma Scale score: 15 (E4V5M6); respiratory rate: 20 breaths/min; pulse rate: 86 beats/min, blood pressure: 194/64 mmHg; and oxygen saturation: 99% via 10 L/min through a reservoir mask. Burn wounds were distributed on his face, neck, anterior trunk, and both upper extremities. Total body surface area (TBSA) affected was 35, 30% with third-degree burns and 5% with deep dermal burns (Fig. 1). As the patient had extensive burns, including on his face and neck, he was immediately intubated and fluid resuscitation was initiated. The burned areas were gently scrubbed with soapy water and covered with non-adherent dressing. The patient was admitted to the emergency intensive care unit.

Silver-coated dressings were applied 2 days after the injury to prevent wound infection. As *Aeromonas hydrophila* was isolated in the wound culture on day 5, 4.5 g doses of piperacillin/tazobactam three times daily were initiated. The medications were continued to treat *Pseudomonas aeruginosa,* which was isolated in the wound on day 7. Tangential debridement and skin grafting were performed for 30% of his TBSA on day 7. The procedure was successfully completed in 3 h.

**Fig. 2:**
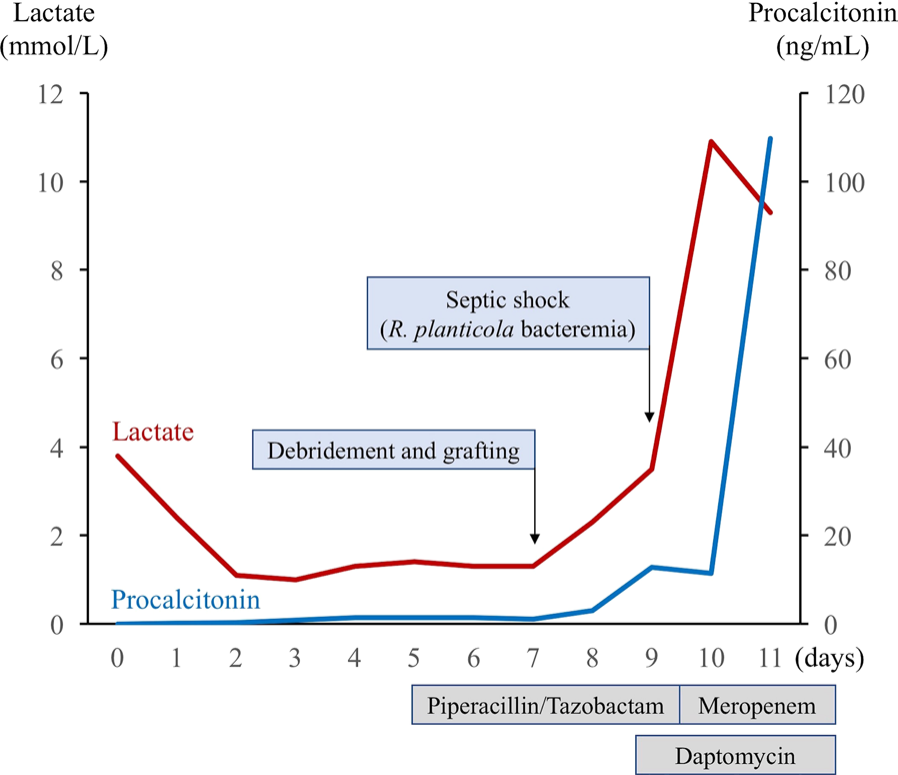
The patient’s clinical course over the 12 days after admission

Two days after surgery, the patient developed septic shock. White blood cell counts showed 33,470/μL with 97.7% neutrophils. His lactate and procalcitonin levels were found to be increased (Fig. 2). Noradrenaline infusion was initiated to support blood pressure following adequate fluid resuscitation. Empirical antibiotic therapy with daptomycin was administered and two sets of blood cultures were obtained. His abdomen was soft and not distended, without any evidence of intra-abdominal infections including cholecystitis or bowel necrosis based on laboratory data and radiological findings. The patient developed refractory septic shock despite the addition of vasopressin. Piperacillin/tazobactam was replaced by 1 g doses of meropenem three times daily for concerning antibiotic resistance on day 10; however, the patient’s condition continued to deteriorate. His blood culture was positive for *R. planticola*, which was identified using the VITEK-2 biochemical identification system. The strain was susceptible to both piperacillin/tazobactam and meropenem. The results of antimicrobial susceptibility testing are summarized in Table 1. Cultures of the patient’s urine and sputum at the onset of septic shock were revealed to be negative. Repetitive burn wound cultures yielded *P. aeruginosa*, which was sensitive to both piperacillin/tazobactam and meropenem with minimum inhibitory concentration (MIC) of ≦ 2 μg/mL and MIC ≦ 0.5 μg/mL, respectively, and methicillin-sensitive *Staphylococcus aureus* (MSSA). The patient ultimately expired due to refractory septic shock and subsequent multiple organ failure on day 12. Figure 2 shows the patient’s clinical course over the 12 days after admission.

**Table 1:** Results of antimicrobial susceptibility testing (μg/mL)

Antimicrobial agent	MIC
Cefazolin	> 16
Ampicillin/sulbactam	8
Ceftriaxone	≦ 0.5
Cefepime	≦ 0.5
Gentamicin	≦ 1
Levofloxacin	≦ 0.25
Imipenem	≦ 0.5
Meropenem	≦ 0.5
Piperacillin/tazobactam	≦ 2
Trimethoprim/sulfamethoxazole	≦ 5

## Discussion

This is the first report of fatal septic shock due to *R. planticola* bacteremia after flame burn injury. Extinguishing a fire with contaminated water can cause infection, and an impaired immune response after extensive burn injury may be responsible for the development of fatal septic shock.

*Raoultella planticola* is a Gram-negative, non-motile, encapsulated bacillus previously classified in the genus *Klebsiella,* including the species *K. planticola* and *K. trevisanii* [4]. In 1986, both microorganisms were combined as *K. planticola* because of deoxyribonucleic acid similarities [5]. The new genus *Raoultella* was established in 2001 on the basis of 16S rRNA and *rpoB* sequences [4]. *R. planticola* is considered an environmental organism commonly isolated from water and soil and does not typically cause human infections [6]. In recent years, infections due to *R. planticola* have been reported, including necrotizing fasciitis, bacteremia, severe pancreatitis, cholecystitis, cholangitis, urinary tract infection, and pneumonia [1, 6–11]. These infections mainly occur in immunocompromised patients with conditions such as cancer and hematological malignancies, and those post-transplant and/or with comorbidities including diabetes and alcoholic cirrhosis [1, 2, 8].

Several infection routes are possible in our case. Although the burn wound culture had been negative for *R. planticola*, the burn wound may have been a possible infection route, considering that *A. hydrophila*, the most common cause of wound infection related to a history of body immersion in untreated water [3], had been identified initially. In addition, both *R. planticola* and *A. hydrophila* are deemed uncommon organisms isolated from outbreaks in burn patients [12]. O’Connell et al. [13] described a case of soft tissue infection caused by *R. planticola* in a healthy individual 1 week after injury to the thumb in a soiled environment. Otherwise, since the patient had plunged into a tank of contaminated water to extinguish the fire on his body, including his face, it was speculated that the oral or gastrointestinal routes might also have contributed to the infection. Lam et al. [6] reported a case of *R. planticola* infection 4 days after a patient with metastatic cancer consumed seafood. Furthermore, extensive burn injury is well-known to lead to host immune system impairment, rendering the patient susceptible to opportunistic infections [14]. Also, escharotomy and skin grafting may be predisposed to bacteremia, as previous reports have suggested the potential risk factors of *R. planticola* bacteremia in invasive medical procedures [9].

Clinical outcomes of *R. planticola* infections have been fair unless polymicrobial bacteremia was present [2, 15]. Chun et al. summarized 20 cases of *R. planticola* bacteremia, six of which were polymicrobial infections; half of those failed to recover [2]. Even though *R. planticola* as a sole pathogen had been isolated from blood cultures in the present case, *P. aeruginosa* and MSSA burn wound infections may accelerate the patient’s development of refractory septic shock. In the past few years, carbapenem-resistant *R. planticola* cases have been reported sporadically, resulting in unfavorable outcomes [15, 16]. In the present case, empiric administration of piperacillin/tazobactam had been initiated prior to the development of septic shock and isolated organisms were susceptible to piperacillin/tazobactam and meropenem. As limited data is available regarding this pathogen and its virulence, the mechanisms of its pathogenesis in the present case with poor outcomes remained unclear. We cannot assert whether the clinical course was associated with *R. planticola* bacteremia; however, it may have played a critical role in the development of fatal septic shock. Further research regarding its virulence is necessary.

The effects of prophylactic antibiotics use for patients with severe burns remains controversial, as a small volume and low-quality data is available [17]. However, a recent systematic review suggests that prophylactic antibiotics may be beneficial for patients requiring mechanical ventilation [18]. Although a history of exposure to soil or water is not always essential for *Aeromonas* spp. infection in burn patients [19], a history of extinguishing the fire with dirty water should alert the physician to consider the possibility of infection from *A. hydrophila* and other microorganisms, including *P. aeruginosa* and *Bacillus cereus* [3]. Hence, initial therapeutic antibiotics may contribute to favorable outcomes in such a specific situation.

To the best of our knowledge, this is the first report of fatal septic shock due to *R. planticola* bacteremia, which was an extremely unusual pathogen in a burn patient. Exposure to contaminated water to extinguish a fire may contribute to *R. planticola* infection. In addition, an impaired immune system due to an extensive third degree burn may further progress to refractory septic shock. Initial empirical antibiotics may be beneficial for burn patients with aquatic exposure.

## Conclusions

We reported a rare case of fatal septic shock due to *R. planticola* bacteremia following flame burn injury. Clinicians should be aware of such fatal infections when a patient is exposed to contaminated water.

## Abbreviations

TBSA: total body surface area
MIC: minimum inhibitory concentration
MSSA: methicillin-sensitive *Staphylococcus aureus*

## References

[1] Kim SH, Roh KH, Yoon YK, Kang DO, Lee DW, Kim MJ (2012). Necrotizing fasciitis involving the chest and abdominal wall caused by *Raoultella planticola*. BMC Infect Dis.

[2] Chun S, Yun JW, Huh HJ, Lee NY (2014). Low virulence? Clinical characteristics of *Raoultella planticola* bacteremia. Infection.

[3] Ribeiro NF, Heath CH, Kierath J, Rea S, Duncan-Smith M, Wood FM (2010). Burn wounds infected by contaminated water: case reports, review of the literature and recommendations for treatment. Burns.

[4] Drancourt M, Bollet C, Carta A, Rousselier P (2001). Phylogenetic analyses of *Klebsiella* species delineate *Klebsiella* and *Raoultella* gen. nov., with description of *Raoultella ornithinolytica* comb. nov., *Raoultella terrigena* comb. nov. and *Raoultella planticola* comb. nov. Int J Syst Evol Microbiol.

[5] Gavini F, Izard D, Grimont PAD, Beji A, Ageron E, Leclerc H (1986). Priority of *Klebsiella planticol* Bagley, Seidler, and Brenner 1982 over *Klebsiella trevisani* Ferragut, Izard, Gavini, Kersters, DeLey, and Leclerc 1983. Int J Syst Bacteriol.

[6] Lam PW, Salit IE (2014). *Raoultella planticola* bacteremia following consumption of seafood. Can J Infect Dis Med Microbiol.

[7] Alves MS, Riley LW, Moreira BM (2007). A case of severe pancreatitis complicated by *Raoultella planticola* infection. J Med Microbiol.

[8] Ershadi A, Weiss E, Verduzco E, Chia D, Sadigh M (2014). Emerging pathogen: a case and review of *Raoultella planticola*. Infection.

[9] Yokota K, Gomi H, Miura Y, Sugano K, Morisawa Y (2012). Cholangitis with septic shock caused by *Raoultella planticola*. J Med Microbiol.

[10] Skelton WP, Taylor Z, Hsu J (2017). A rare case of *Raoultella planticola* urinary tract infection in an immunocompromised patient with multiple myeloma. IDCases.

[11] Westerveld D, Hussain J, Aljaafareh A, Ataya A (2017). A rare case of *Raoultella planticola* pneumonia: an emerging pathogen. Respir Med Case Rep.

[12] Yun HC, Tully CC, Mende K, Castillo M, Murray CK (2016). A single-center, six-year evaluation of the role of pulsed-field gel electrophoresis in suspected burn center outbreaks. Burns.

[13] O’Connell K, Kelly J, Niriain U (2010). A rare case of soft-tissue infection caused by *Raoultella planticola*. Case Rep Med.

[14] Bohr S, Patel SJ, Vasko R, Shen K, Golberg A, Berthiaume F (2015). The role of CHI3L1 (Chitinase-3-Like-1) in the pathogenesis of infections in burns in a mouse model. PLoS ONE.

[15] Demiray T, Koroglu M, Ozbek A, Altindis M (2016). A rare cause of infection, *Raoultella planticola*: emerging threat and new reservoir for carbapenem resistance. Infection.

[16] Castanheira M, Deshpande LM, DiPersio JR, Kang J, Weinstein MP, Jones RN (2009). First descriptions of blaKPC in *Raoultella* spp. (*R. planticola* and *R. ornithinolytica*): report from the SENTRY Antimicrobial Surveillance Program. J Clin Microbiol.

[17] Avni T, Levcovich A, Ad-El DD, Leibovici L, Paul M (2010). Prophylactic antibiotics for burns patients: systematic review and meta-analysis. BMJ.

[18] Ramos G, Cornistein W, Cerino GT, Nacif G (2017). Systemic antimicrobial prophylaxis in burn patients: systematic review. J Hosp Infect.

[19] Chim H, Song C (2007). Aeromonas infection in critically ill burn patients. Burns.

